# 12/15-lipoxygenase mediates disturbed flow-induced endothelial dysfunction and atherosclerosis

**DOI:** 10.1186/s10020-025-01297-0

**Published:** 2025-07-15

**Authors:** Jia Wei Chen, Shi Li Chen, Xin Rui Wu, Xin Yi Shu, Si Yi Tang, He Yuan, You Ran Li, Jin Wei Quan, Shuo Feng, Rui Yan Zhang, Chen Die Yang, Lin Lu, Xiao Qun Wang

**Affiliations:** 1 Department of Cardiovascular Medicine, Ruijin Hospital, Shanghai Jiao-Tong University School of Medicine, 197 Ruijin Er Rd, Shanghai, 200025 China; 2 Institute of Cardiovascular Diseases, Shanghai Jiao-Tong University School of Medicine, Shanghai, 200025 China; 3 Shanghai Key Laboratory of Biliary Tract Disease Research, Department of General Surgery, Xinhua Hospital, Shanghai Jiao Tong University School of Medicine, Shanghai, 200092 China

**Keywords:** Atherosclerosis, Disturbed flow, Endothelial dysfunction, 12/15-lipoxygenase, Shear stress

## Abstract

**Background:**

Disturbed flow regions in the vasculature are predisposed to endothelial dysfunction and atherosclerotic plaque formation. The enzyme 12/15-lipoxygenase (12/15-LOX, encoded by ALOX15) has emerged as a promising therapeutic target for atherosclerosis. However, the relationship between 12/15-LOX and disturbed flow-induced atherosclerosis remains uncharacterized.

**Methods:**

Expression of 12/15-LOX in endothelial cells (ECs) exposed to steady flow and disturbed flow was compared in vivo and in vitro*.* The effect of 12/15-LOX on ECs was analyzed by using ALOX15 knockout mice, EC-specific adeno-associated virus (AAV)-mediated delivery of ALOX15-shRNA, and specific inhibitors. Partial carotid ligation mouse model was established to ascertain the role of 12/15-LOX in ECs under disturbed flow.

**Results:**

Compared to steady flow regions, 12/15-LOX was significantly upregulated in ECs at disturbed flow sites. In vivo and in vitro experiments demonstrated that 12/15-LOX promoted disturbed flow-elicited endothelial dysfunction. Mass spectrometry analysis revealed that 12/15-LOX promoted production of 15 s-HETE, a pro-inflammatory eicosanoid metabolite, in ECs exposed to disturbed flow. Furthermore, we showed that disturbed flow activated 12/15-LOX expression through transactivation of its promoter by a mechanosensitive transcription factor sterol regulatory element binding protein 2 (SREBP2). Finally, EC-specific knockdown or inhibition of 12/15-LOX substantially attenuated the development of atherosclerosis in disturbed flow regions.

**Conclusions:**

Disturbed flow promoted 12/15-LOX expression via SREBP2, thereby leading to increased pro-inflammatory PUFA metabolites and ECs dysfunction. Targeting at SREBP2-12/15-LOX pathway should provide therapeutic perspectives to attenuate disturbed flow-induced atherosclerosis.

**Graphical abstract:**

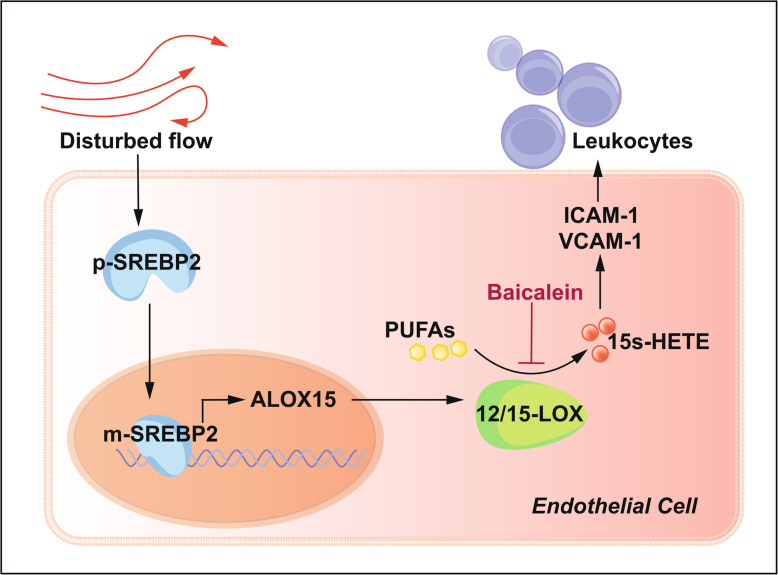

**Supplementary Information:**

The online version contains supplementary material available at 10.1186/s10020-025-01297-0.

## Introduction

Hemodynamic patterns are not uniform in the vascular system, with straight vessel regions experiencing steady flow characterized by high unidirectional wall shear stress, while arterial branches and curvatures are exposed to disturbed flow characterized by low, oscillatory shear stress. These hemodynamic patterns critically regulate vascular homeostasis, with disturbed flow promoting atherosclerosis (Chiu, et al. 2011, Kwak, et al. 2014). Endothelial cells (ECs) behave differentially under different flow profiles by sensing specific shear stress patterns and transducing mechano-signals to regulate biological functions(Niu, et al. 2019). Local endothelial disturbance by disturbed flow, together with systemic risk factors, such as dyslipidemia and diabetes, synergistically promote the pathogenesis of atherosclerosis, plaque rupture, and acute coronary events (Brown, et al. 2016, Koskinas, et al. 2013). Although the predictability of disturbed flow regions using computational fluid dynamics (Brown, et al. 2016), the molecular mechanisms linking hemodynamics to atherosclerosis remain incompletely understood, limiting the development of new therapeutic strategies.

Eicosanoids, a group of bioactive lipids derived from oxidative modification of arachidonic acids or other polyunsaturated fatty acids (PUFA), are consistently observed in atherosclerotic lesions (Folcik, et al. 1995, Mallick, et al. 2022, Sherratt, et al. 2024). A number of enzymes are implicated in the oxidative degradation of arachidonic acids to produce eicosanoid metabolites, including cyclooxygenase (COX), lipoxygenase (LOX) and cytochrome P450 (CYP). In pathological conditions, maladaptive modulation of these enzymes and the resultant dysregulation of lipid mediators contribute to inflammatory response and plaque progression (Biringer 2020, Capra, et al. 2013, Piper, et al. 2020). Among these enzymes, 12/15-LOX—the murine ortholog of human 15-LOX—has emerged as a critical player in atherogenesis (George, et al. 2001, Singh, et al. 2019). This enzyme is encoded by ALOX15 gene and oxidizes PUFAs in a stereospecific manner to generate a profile of eicosanoid metabolites, such as 12/15S-hydroxy-5Z,8Z,10E,14Z-eicosatetraenoic acid (12/15 s-HETE) and 9/13S-hydroxy-10E,12Z-octadecadienoic acid (9/13 s-HODE) (Singh and Rao 2019). Extensive studies have demonstrated the involvement of 12/15-LOX in endothelial activation, monocyte recruitment, and macrophage-mediated lipoprotein oxidation, all of which are key processes in atherosclerotic plaque development (Bolick, et al. 2006, Cyrus, et al. 2001, Cyrus, et al. 1999, George, et al. 2001, Harats, et al. 2000, Huo, et al. 2004, Singh and Rao 2019). However, the role of 12/15-LOX and its eicosanoid metabolites in ECs in response to disturbed flow remains undetermined.

In this study, we discovered a significant increase of endothelial 12/15-LOX expression in disturbed flow regions through transcriptive activation of its promoter by SREBP2. We revealed that 12/15-LOX enhanced disturbed flow-elicited EC inflammation via promoting eicosanoid metabolites production. We demonstrated that genetic or pharmacologic inhibition of 12/15-LOX substantially attenuated disturbed flow-induced atherosclerosis. By exploring the interplay between hemodynamic forces and EC inflammation, we seek to uncover novel therapeutic strategies targeting 12/15-LOX for the prevention and treatment of atherosclerosis.

## Methods

Detailed experimental methods and materials are provided in Supplemental Material.

### Human arterial specimens

This study complied with the Declaration of Helsinki. Human internal mammary arteries were obtained from patients undergoing coronary artery graft with informed consents, and approval by Ethics Committee of Ruijin Hospital, Shanghai Jiao-Tong University School of Medicine. Detailed information is provided in the Supplemental Material.

### Animals

Mice with global ALOX15 gene deletion (C57BL/6 background) were purchased from The Jackson Laboratory (Bar Harbor, Marine, USA). C57BL/6 and ApoE^−/−^ mice were purchased from Charles River Laboratory Animal Technology Co., Ltd (Beijing, China). Then ALOX15^−/−^ mice were crossed with ApoE^−/−^ mice to generate ApoE^−/−^/ALOX15^−/−^ mice, as previously described (Li, et al. 2018). Control mice with the same age and sex from littermates were used in experiments involving in ALOX15^−/−^ mice. To evaluate the potential role of 12/15-LOX in the vascular endothelium of mice, we established AAV-mediated delivery systems expressing either ALOX15 specific shRNA or scramble control shRNA, both driven by the endothelial cell-specific Cdh5 promoter. The constructs, pAAV-Cdh5-EGFP-3xFLAG-miR30 shRNA (ALOX15)-tWPA and its control virus, were packaged into AAV particles for in vivo delivery. Virus (5 × 10^11^ gc/mice) were delivered to ApoE^−/−^mice (male, 8 weeks old) via tail vein injection. 3 weeks after injection, the efficiency of virous was evaluated and mice were subjected to carotid artery partial ligation or sham surgery and high fat diet for 2 weeks. To examine the pharmacological and therapeutic effects of baicalein, ApoE^−/−^ mice were intraperitoneally injected with baicalein (20 mg/kg/day) 2 days before partial carotid ligation and continued until euthanasia. To establish animal models, mice anesthesia was induced by inhalation of 3–5% isoflurane and then maintained by 1–2% isoflurane with a small rodent respirator during surgery. For euthanatizing, mice were anesthetized with intraperitoneal injection of sodium pentobarbital (50 mg/kg) and heparin and euthanatized by cervical dislocation. The mice were acclimated to the laboratory environment for one week before experimentation.

All animal procedures were performed in accordance with the National Institutes of Health (NIH) and approved by Ethics Committee of Ruijin Hospital, Shanghai Jiao-Tong University School of Medicine.

### Partial ligation surgery and vascular ultrasonography

LCA partial ligation surgery were performed on mice, as described in reference (Nam, et al. 2009). Briefly, after mice anesthesia was induced as mentioned above, a midline ventral incision (8–10 mm) was made in neck epilated area. Muscles in carotid triangles were bluntly dissected to expose LCA (left common carotid artery) and RCA (right common carotid artery). Three distal branches of LCA (occipital artery, internal carotid and left external carotid artery) were tightly ligated by 6–0 silk suture, with left superior thyroid artery remaining intact. After ligation, the incision was sutured with 3–0 silk suture. Measures were taken to maintain mice body temperature, pain relief, and infectious prevention. C57BL/6 mice was fed with chow diet for 2 weeks post-operation. Mice with ApoE^−/−^ background were fed with high fat diet (40 kcal% fat, 1.25% cholesterol, 0.5% cholic acid, cat# D12109, Research Diets, Inc., New Brunswick, NJ) from the day after surgery to 2 weeks. Echography was performed before surgery, 1 day and 2 weeks after surgery to measure common carotid arteries flow velocity in pulse wave doppler mode. All ultrasound measurements were taken in anesthetized mice with Vevo 2100 high-resolution micro-ultrasound imaging system (Toronto, Canada).

### Intravital microscopy

Mice were anesthetized as mentioned above. Cremaster muscle was isolated, and arterioles were observed by microscope. Rolling and adhesive leukocytes labeled by 0.4 mg/ml Rhodamine 6G via retro-orbital injection were then monitored and recorded. Fiji ImageJ were used to analyze the numbers of rolling and adhesive leukocytes in arterioles of certain regions in a certain period.

### Cell culture and application of shear stress in vitro

HUVECs were cultured in endothelial cell medium (ECM) with 5% fetal bovine serum (FBS), 100 µg/ml streptomycin and 100 IU/ml penicillin in humidified cell culture incubator (37 °C, 95% air and 5% CO_2_). THP-1 were cultured in RPMI medium with 10% FBS, 100 µg/ml streptomycin and 100 IU/ml penicillin in the same environment as HUVECs.

IBIDI flow pump system and an orbital shaker were used to generate steady flow or disturbed flow on HUVECs. HUVECs were seeded on IBIDI µ-slides coated with adhesion factor. The slide was connected to the IBIDI perfusion set when cells at 100% confluence. For steady flow, HUVECs were exposed to shear stress of 13 dyn/cm^2^. For disturbed flow, cells were exposed to shear stress of ± 5 dyn/cm^2^.

To collect a large amount of cells for biochemical analysis, HUVECs plated on 6-well plate were set up into an orbital shaker that sheared at 180 rpm (~ 14 dyn/cm^2^) for steady flow or at ± 90 rpm (± 5 dyn/cm^2^) for disturbed flow, as described previously (Liu, et al. 2017). The shear stress imposed on the HUVEC was calculated by the formula $\alpha \sqrt{{\eta \rho (2\pi f)}^{3}}$ where α is the radium of rotation, η is the viscosity of medium, ρ is the density of medium, $f$ is the rotation speed. To generate steady flow by the orbital shaker, cells were segmented by a patch of Pluronic F-127 solution as described by Fernandes (Fernandes, et al. 2022) and Ghim (Ghim, et al. 2018) with modification. Briefly, to prevent cells growth in the area of disturbed flow, 6-well plates were coated with 1% Pluronic F-127 solution from the center to radius of 7.84 mm in the bottom of the wells. After one hour coating and rinse with PBS, HUVECs were plated and grew to confluence only in the area not coated with the 1% Pluronic F-127 solution. Afterwards, we harvested cells in the outer area under exposure to steady flow. To keep consistency, cells treated with disturbed flow were also cultured in such pretreated plates and thus only grew in the outer area.

### Immunofluorescence

Immunofluorescence staining of human coronary arteries and *en face* immunofluorescence staining of mouse aorta were performed as detailed in the Supplemental Material as previously described(Li, et al. 2019, Wang, et al. 2012).

### Histology

Oil Red O (ORO) staining and Hematoxylin and eosin staining were performed as detailed in the Supplemental Material.

### Real time quantitative polymerase chain reaction (PCR)

Real time-PCR was performed as detailed in the Supplemental Material.

### Western blot analysis

Western blot was performed as detailed in the Supplemental Material.

### LC–MS/MS-based eicosanoids analysis

Eicosanoid mediators were extracted from 5 × 10^6^ HUVECs subjected to steady flow or disturbed flow with 1 mL methanol/water solution (v/v = 4:1) containing (d4) 13-HODE, (d4) 9-HODE, (d8) 15-HETE, (d8) 12-HETE and (d8) 5-HETE as the internal standards. After cell disruption and sonication for 5 min by a Bioruptor® Plus device (DIAGENODE S.A., BELGIUM), protein was precipitated in 4 °C for 1 h and then removed by centrifugation (12,000 rpm, 4 °C, 15 min). The upper-layer extract of each sample was vacuum-dried by a Labconco Vacuum Concentrator (Kansas, MO, USA) and then stored at −80 °C before LC–MS/MS analysis.

The dry extracts were dissolved in 100 mL of methanol/water solution (v/v = 1:4) and 10 mL of the dissolved extract was injected for subsequent LC–MS/MS analysis. The eicosanoid analysis was carried out on a TSQ Quantiva Triple Quadrupole Mass Spectrometer equipped with an Ultimate 3000 UHPLC system (Thermo, MA, USA) according to the published method (Chen, et al. 2019)with modification. Eicosanoids were separated by an Acquity UPLC BEH C18 column (1.7 mm, 2.1 × 100 mm, Waters, MA, USA) maintaining at 40 °C. The mobile phase A was 0.1% formic acid in water, and mobile phase B was 0.1% formic acid in acetonitrile. The flow rate was 0.3 mL/min, and the elution gradient was as follows: 0—0.5 min, 30% B; 1.0—2.5 min, 40% B; 4.5—6.5 min, 70% B; 9.0—12.0 min, 95%; 12.1—15.0 min, 30% B.

The MS with electrospray ionization was operated in negative ion mode with scheduled multiple reaction monitoring (MRM) for detection of eicosanoids and internal standards. Spray voltage was set at −2500 V. The sheath gas, aux gas, sweep gas, ion transfer tube and vaporizer temperature were set at 40 Arb, 8 Arb, 1Arb, 350 °C and 350 °C, respectively. The optimized MRM transitions and their respective collision energies were listed in Supplementary Data.

Thermo Xcalibur Workstation and Quan Browser were employed for MRM data acquisition and processing. Quantification is based on the peak area ratio of measured eicosanoids and their corresponding internal standards with defined concentration.

### Bioinformatics analysis

Transcription factor binding sites prediction was based on JASPAR database. The promoter region was defined as upstream of 2000 bp to the transcriptional start site of ALOX15 gene.

### Dual luciferase assay

Dual luciferase assay was performed as detailed in the Supplemental Material.

### Chromatin immunoprecipitation (ChIP)

Chromatin immunoprecipitation was performed using commercial kit with modification as detailed in the Supplemental Material.

### Statistical analysis

Date is presented as mean ± SEM or median with interquartile range based on data distribution. Shapiro–Wilk test was used to test for data normality. Levene test was used to test for the equality of group variances. Proper post hoc tests were used to correct for multiple comparisons. Two-sided tests at 5% level of significance were used in all parametric and nonparametric tests. Detailed statistical methods were mentioned in corresponding figure legends. All statistical analyses were conducted using GraphPad Prism 9.0 or SPSS 26.0.

## Results

### 12/15-LOX expression is increased in ECs in response to disturbed flow

**Fig. 1:**
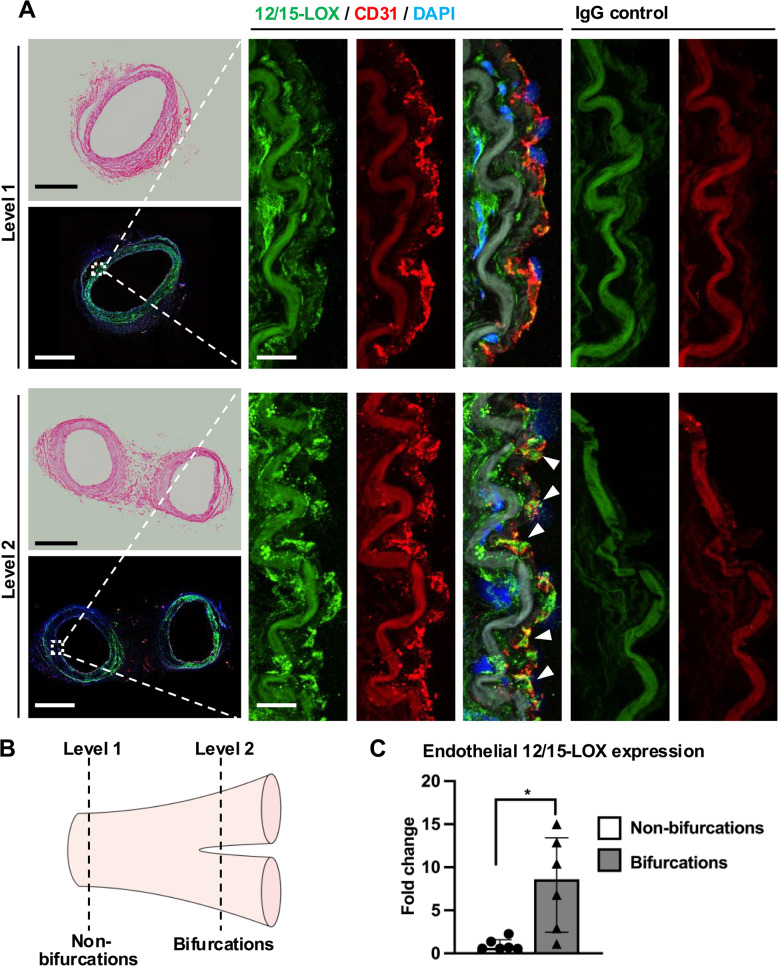
12/15-LOX expression is increased in ECs in disturbed flow regions of human arteries. **A** Representative images of H&E staining and immunofluorescence staining of 12/15-LOX and CD31 in steady flow region (straight part, non-bifurcations) or disturbed flow regions (bifurcations) of human internal mammary arteries. Dashed rectangles indicate arterial intima. Scale bar, 1000 μm at low magnification, 20 μm at high magnification. **B** A schematic diagram of artery with bifurcations. Level 1 indicates the straight part of artery that is under steady flow, level 2 indicates bifurcations under disturbed flow. **C** Quantification of 12/15-LOX expression in arterial intima (*n* = 6). Data are expressed as median with interquartile range (**C**). Statistics: Wilcoxon test in (**C**). * *P* < 0.05

To investigate the impact of shear stress on 12/15-LOX expression, human intramammary arteries with bifurcations were transversely sectioned at levels exposed to different flow profiles. Compared to the straight part of the vessel under steady flow (level 1), 12/15-LOX was markedly upregulated in ECs near outer walls of the bifurcation exposed to disturbed flow (level 2) (Fig. 1A-C). Meanwhile, although 12/15-LOX was also detected in the media of the artery, no difference in 12/15-LOX expression was observed among different levels.

**Fig. 2:**
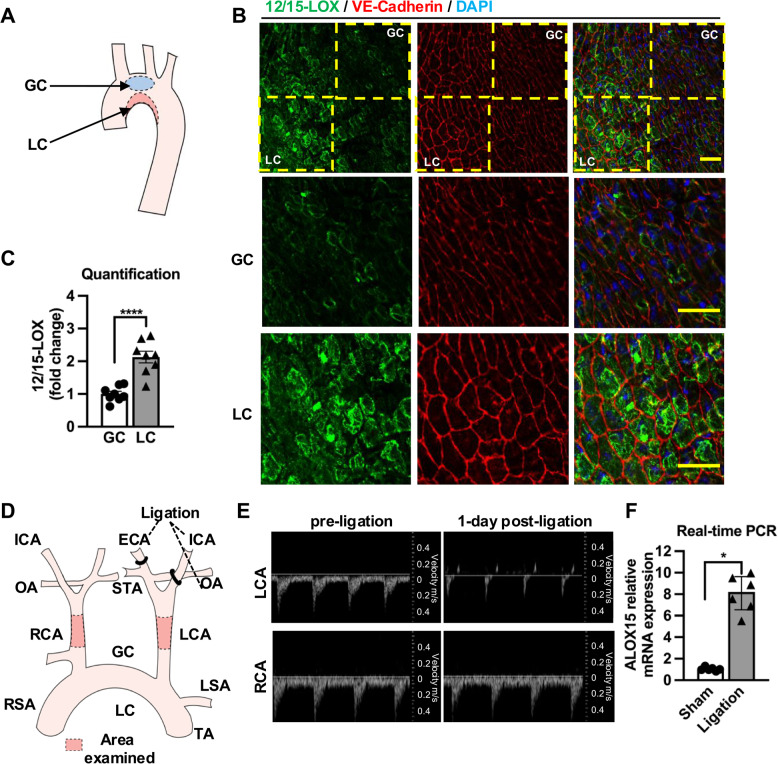
12/15-LOX expression is increased in ECs in disturbed flow regions of murine arteries. **A** A schematic diagram of aortic arch. GC indicates the great curvature. LC indicates the lesser curvature. **B-C** Representative images of en face immunofluorescence staining of 12/15-LOX and VE-cadherin expression in GC or LC of mouse aortic arch were shown and (**C**) quantified (*n* = 8). Arrow heads indicate colocalization of 12/15-LOX and VE-cadherin. Scale bar, 30 μm. **D** A schematic diagram of carotid artery partial ligation surgery. **E** Representative M-mode ultrasonographic images showing flow velocity in mouse right (RCA) and left common carotid (LCA) arteries after sham or partial ligation surgery. **F** Real-time PCR were performed to quantify ALOX15 mRNA levels in intima of common carotid artery after surgery for 2 weeks, with normalization to GAPDH (*n* = 6). Data are expressed as the mean ± SEM (**C**) or median with interquartile range (**F**). Statistics: Paired t test in (**C**). Wilcoxon test in (**F**). * *P* < 0.05, ****P < 0.0001

Consistently, *en face* staining of mouse aorta (Fig. 2A) showed that 12/15-LOX was significantly increased in ECs in the lesser curvature (disturbed flow regions) compared with the great curvature (steady flow regions) of the aortic arch (Fig. 2B and C). Further analysis revealed that 12/15-LOX expression was upregulated in the plasma membrane, cytosol and nucleus in ECs in the disturbed flow regions (Supplementary Fig. 1A-F). Furthermore, partial ligation of mouse carotid artery (Nam, et al. 2009, Souilhol, et al. 2020) was performed to induce local disturbed flow proximal to the ligation site (Fig. 2D and E). Real-time PCR revealed that ALOX15 mRNA level was significantly increased in the intima of the partially ligated carotid artery compared to the sham operated controls (Fig. 2F).

Next, human umbilical vein endothelial cells (HUVECs) were treated with disturbed flow or steady flow in vitro. We found ALOX15 mRNA (Supplementary Fig. 2A), and 12/15-LOX and ICAM-1 protein (Supplementary Fig. 2B-C) expression of 12/15-LOX were increased in response to disturbed flow in a time-dependent manner.

### Inhibition of 12/15-LOX attenuates disturbed flow-elicited EC dysfunction

**Fig. 3:**
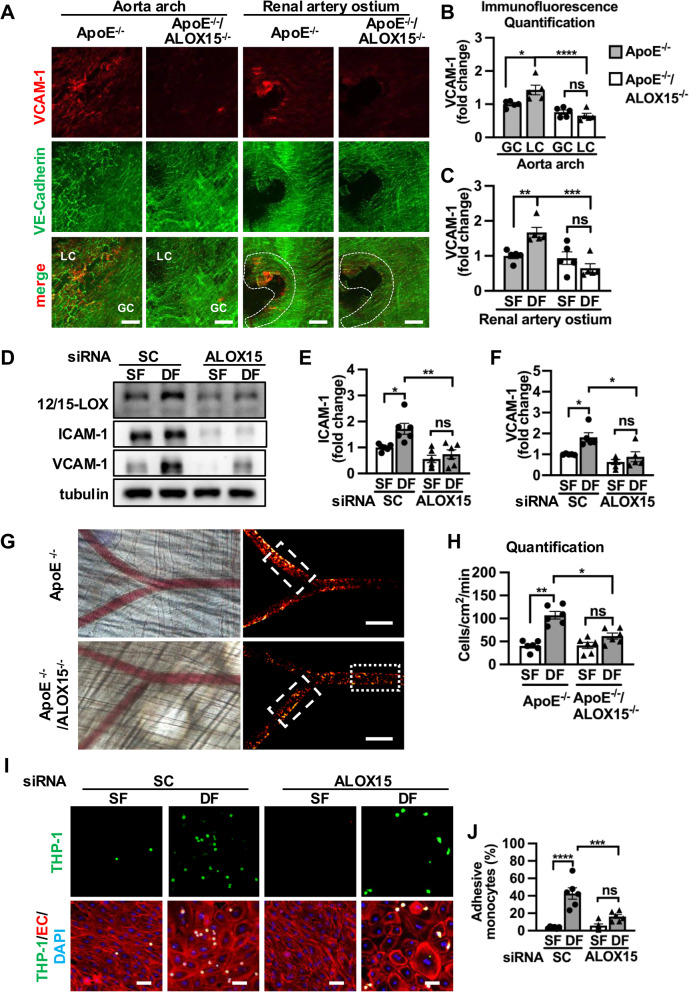
12/15-LOX deficiency attenuates disturbed flow-elicited EC inflammation. **A-C** Representative images of en face immunofluorescence staining of 12/15-LOX and VE-cadherin expression in aortic arch and renal artery ostium from ApoE^−/−^ and ApoE^−/−^/ALOX15^−/−^ mice. GC and LC denote the great curvature and the lesser curvature of aortic arch, respectively; Dashed area indicates renal artery ostium. Scale bar, 50 μm. **B-C** Bar graph shows quantification of VCAM-1 expression by immunofluorescence staining (*n* = 5). **D-F** HUVECs transfected with scramble (SC), or ALOX15 siRNA were exposed to either steady flow or disturbed flow for 24 h. Protein levels of 12/15-LOX, ICAM-1, VCAM-1 were analyzed by Western Blot and (**E–F**) quantified (*n* = 5–6). **G-H** Representative heatmaps show overlaying images over a 10 s period (57 frames/second) of leukocytes marked by Rhodamine 6G staining in bifurcated arteries of cremaster muscle in ApoE^−/−^ and ApoE^−/−^/ALOX15^−/−^ mice. Original recorded videos were processing by ImageJ software. Dotted rectangles indicate steady flow regions, dashed rectangles indicate disturbed flow region. Scale bar, 100 μm. **H** Bar graph shows the number of rolling leukocytes (*n* = 6). **I-J** HUVECs transfected with scramble (SC), or ALOX15 siRNA were exposed to either steady flow or disturbed flow for 24 h, and then co-incubated with THP-1 monocytes labeled by Calcein-AM. **J**Adhesive THP-1 monocytes remained after PBS rinse and were quantified as percentage of THP-1 monocytes to HUVECs (*n* = 6). Scale bar, 50 μm. Data are expressed as the mean ± SEM (**B**, **C**, **E**, **G**, **H**, **J**). Statistics: 2-way ANOVA with Holm-Šídák’s post hoc test for 3 comparisons in (**E**, **F** and **J**). Repeated measures ANOVA with Holm-Šídák’s post hoc test for 3 comparisons in (**B**, **C** and **H**). *P < 0.05, **P < 0.01, ***P < 0.001, ****P < 0.0001. ns, no significant difference

The role of 12/15-LOX in endothelium under disturbed flow was tested. *En* face immunofluorescence staining demonstrated that upregulation of VCAM-1 under disturbed flow was attenuated in ALOX15 knockout mice compared to wildtype controls (Fig. 3A-C). Consistently, disturbed flow-induced VCAM-1 and ICAM-1 in cultured ECs were suppressed by 12/15-LOX knockdown (Fig. 3D-F). Overexpression of 12/15-LOX promoted ICAM-1 expression in ECs under steady flow, and upregulation of ICAM-1 was more prominent under disturbed flow than steady flow (Supplementary Fig. 3A-B), indicating that 12/15-LOX overexpression sensitizes ECs to dysfunction status more under disturbed flow. Next, 12/15-LOX activity was inhibited by a pharmacological inhibitor baicalein. We found inhibiting 12/15-LOX in ECs reduced upregulation of VCAM-1 and ICAM-1 under disturbed flow (Supplementary Fig. 4A-C). Moreover, leukocyte-endothelium interactions in areas under different flow patterns were compared by intravital microscopy of cremaster arterioles. In contrast to steady flow areas, leukocytes rolling and adhesion to endothelium were significantly increased in disturbed flow areas in ApoE^−/−^ mice. However, increased rolling and adhesive leukocytes on endothelium in disturbed flow regions were reversed in either ALOX15 ^−/−^/ApoE^−/−^ mice (Fig. 3G and H) or ApoE^−/−^ mice with intraperitoneal injection of baicalein (Supplementary Fig. 4D-E). Similarly, in vitro adhesion assay also revealed that inhibition (Fig. 3I, J and Supplementary Fig. 4F-G) of 12/15-LOX suppressed EC-monocyte adhesion under disturbed flow.

### 12/15-LOX upregulates pro-inflammatory eicosanoid metabolite 15 s-HETE under disturbed flow

**Fig. 4:**
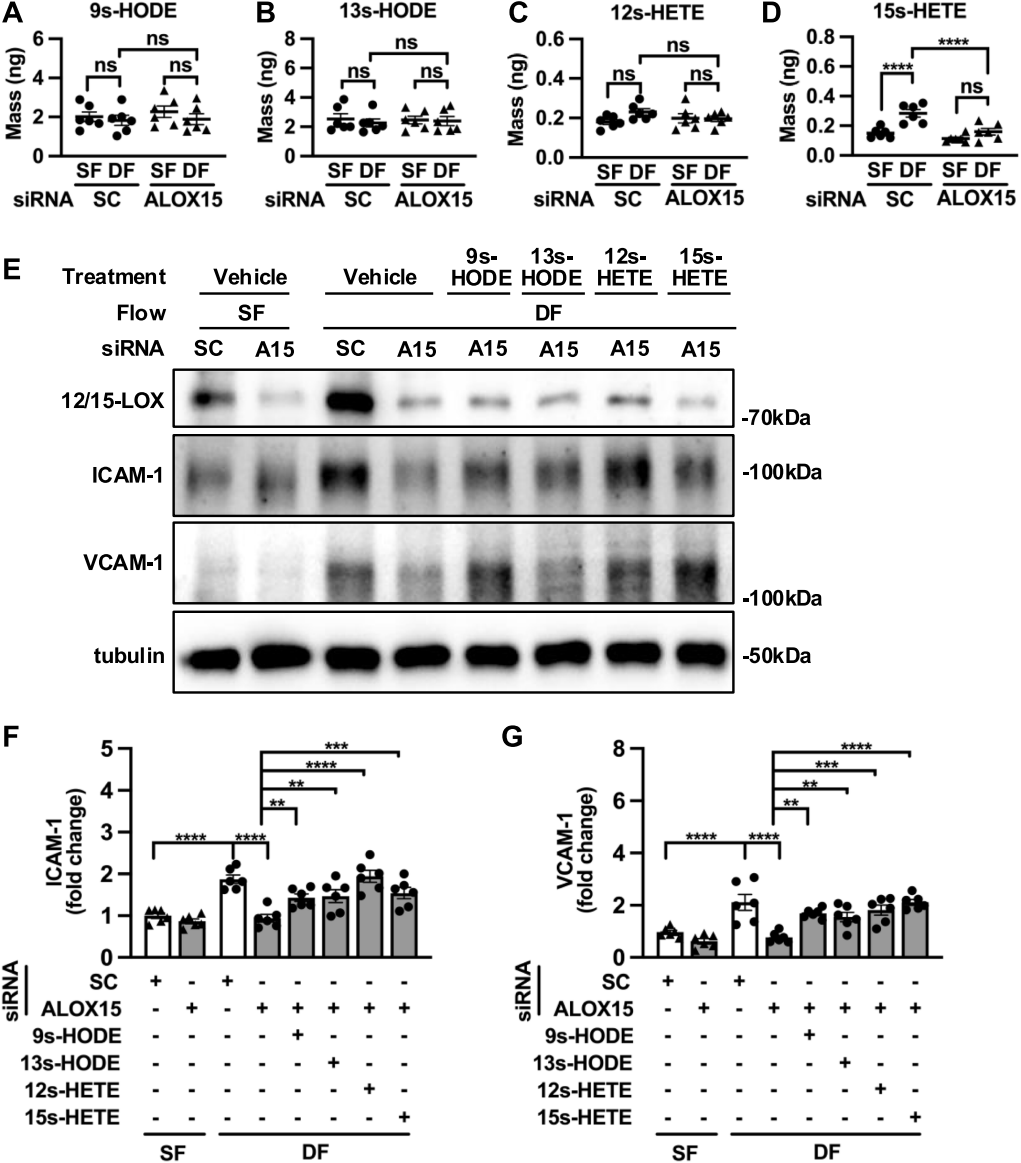
12/15-LOX promotes the production of pro-inflammatory PUFA metabolites. **A-D** HUVECs were transfected with ALOX15 siRNA, followed by exposure to steady flow or disturbed flow for 24 h. 9 s-HODE, 13 s-HODE, 12 s-HETE and 15 s-HETE levels (ng/5 × 10^6^ cells) in HUVECs were determined by LC–MS/MS analysis (*n* = 6). **E**–**G** HUVECs transfected with scramble or ALOX15 siRNA were exposed to steady flow or disturbed flow and rescued by treatment of 9 s-HODE (5 μM), 13 s HODE(5 μM), 12 s-HETE (250 nM), and 15 s-HETE (250 nM), respectively. Protein levels of 12/15-LOX, ICAM-1, VCAM-1 were analyzed and (**F**-**G**) quantified by Western Blot (*n* = 6). Data are expressed as the mean ± SEM (**A** through **G**). Statistics: 2-way ANOVA with Holm-Šídák’s post hoc test for 3 comparisons in (**A** through **D**), and 6 comparisons in (**F** and **G**). **P < 0.01, ***P < 0.001, ****P < 0.0001. ns, no significant difference

To explore whether 12/15-LOX promotes EC dysfunction through its eicosanoid products, we analyzed levels of 12/15-LOX products in ECs by liquid chromatography-mass spectrometry (LC–MS/MS). Compared to cells under steady flow, the levels of 15 s-HETE were markedly increased in ECs subjected to disturbed flow, while levels of 9 s-HODE, 13 s-HODE and 12 s-HETE were comparable under different flow patterns (Fig. 4A-D). Disturbed flow-induced upregulation of 15 s-HETE was attenuated either by 12/15-LOX deficiency (Fig. 4A-D) or pharmacological inhibition with baicalein (Supplementary Fig. 5A-D). Furthermore, abrogation of disturbed flow-elicited ICAM-1 and VCAM-1 in 12/15-LOX-depleted ECs was reversed by exogenous administration of 12/15-LOX PUFA metabolites (Fig. 4E-G).

To determine whether inhibiting 12/15-LOX leads to compensatory flux of arachidonic acid-derived metabolites, the levels of metabolites of COX or CYP were measured. Compared to steady flow, disturbed flow increased the levels of endothelial 6κPGF1α (Supplementary Fig. 6 A), a COX2-derived product known to be regulated by shear stress (Mitchell, et al. 1979, Russell-Puleri, et al. 2017), irrespective of the expression of 12/15-LOX. Other inflammation-associated metabolic products, such as PGE2 and PGF2α (Aoki, et al. 2012, Arnould, et al. 2001) (Supplementary Fig. 6B-C), were also unaffected by 12/15-LOX knockdown under either flow condition.

### SREBP2 regulates disturbed flow-induced 12/15-LOX transcription

**Fig. 5:**
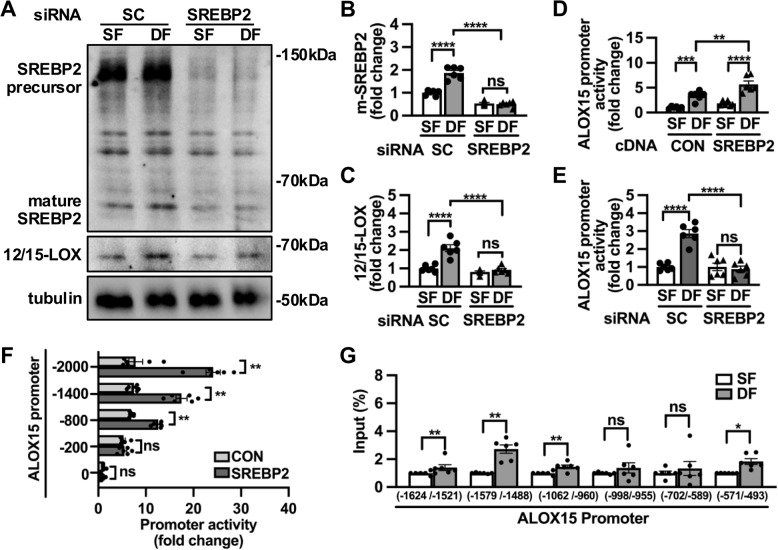
SREBP2 regulates disturbed flow-induced ALOX15 transcription. **A-C** HUVECs transfected with scramble or SREBP2 siRNA were exposed to steady flow or disturbed flow. Protein levels of mature SREBP2 and 12/15-LOX were analyzed by Western Blot and (**B-C**) quantified (*n* = 6). **D**-**E** ALOX15 promoter activity were measured in HUVECs transfected with (**D**) SREBP2 plasmid or (**E**) SREBP2 siRNA with steady flow or disturbed flow exposure (*n* = 6). **F** Full length and serial truncated fragments of ALOX15 promoter activity were compared in 293 T cells transfected with control or SREBP2 plasmid (*n* = 6). **G** ChIP-qPCR assay was performed for SREBP2 occupancy at ALOX15 promoter in HUVECs after exposure to steady flow or disturbed flow (*n* = 6). Data are expressed as the mean ± SEM (**B** through **G**). Statistics: 2-way ANOVA with Holm-Šídák’s post hoc test for 3 comparisons in (**B** through **E**); Unpaired t test, Welch's t test, Mann–Whitney test in (**F** and **G**) * *P* < 0.05, **P < 0.01, ***P < 0.001, ****P < 0.0001. ns, no significant difference

Bioinformatics predicted the presence of sterol regulatory element (SRE) binding sites in the promoter of human ALOX15 gene (Supplementary Fig. 7 A). SREBP2 is a shear stress-sensitive transcriptional factor by interacting with SRE (Xiao, et al. 2013). In endothelium where flow is disturbed, SREBP2 precursor is cleaved to mature SREBP2, thereby translocating to nuclei and activating transcription of genes involved in oxidative stress and inflammation (Xiao, et al. 2013).We observed increased production of mature SREBP2 in ECs under disturbed flow (Fig. 5A and B). After depletion of SREBP2 with the specific siRNA, disturbed flow-induced upregulation of 12/15-LOX was abolished (Fig. 5A and C). These results suggest that SREBP2 is required for disturbed flow-induced 12/15-LOX expression. To determine whether 12/15-LOX was transactivated by SREBP2, HUVECs were transfected with luciferase reporters driven by full-length or serial truncated fragments of the ALOX15 promoter. We found disturbed flow significantly increased the activity of ALOX15 promoter in ECs compared to steady flow, which was further enhanced after overexpression of SREBP2 (Fig. 5D). Conversely, disturbed flow-induced ALOX15 promoter activation was virtually abolished by SREBP2 depletion (Fig. 5E). Luciferase assay with serial truncated fragments of the ALOX15 promoter revealed that the −800 to −200 bp region of the ALOX15 promoter was required for the transactivation of ALOX15 by SREBP2 under disturbed flow (Fig. 5F). ChIP assay validated increased binding of SREBP2 on SRE sites that contain consensus sequence of GT(G)GGGG(C)TGG(A)T(A) in ALOX15 promoter (Fig. 5G), which is consistent with the bioinformatics analysis (Supplementary Fig. 7 A).

### 12/15-LOX deficiency or inhibition attenuates disturbed flow-activated atherosclerosis susceptibility in mice

**Fig. 6:**
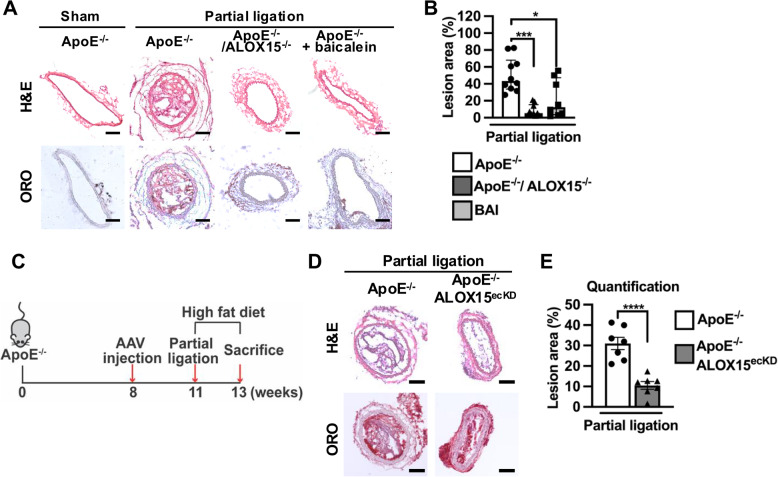
12/15-LOX deficiency or inhibition attenuates disturbed flow-activated atherosclerosis susceptibility in mice. **A-B** ApoE^−/−^, ApoE^−/−^/ALOX15^−/−^ or ApoE^−/−^ mice injected with baicalein (20 mg/kg/day) were subjected to carotid artery partial ligation or sham surgery and high fat diet for 2 weeks. **A** Representative images of ORO, H&E were shown. Scale bar, 100 μm. **B** Percentage of atherosclerotic lesions in the whole cross-sectional arterial area was quantified (*n* = 8–10).** C** Diagram showing experimental protocol. ApoE^−/−^ mice were intravenously injected with AAV-Cdh5-EGFP-Vector or AAV-Cdh5-EGFP-ALOX15 shRNA. 3 weeks later, mice were subjected to carotid artery partial ligation or sham surgery and high fat diet for 2 weeks. **D-E** Representative images of (**D**) ORO and H&E staining in arteries from mice as mentioned in **C**. Scale bar, 50 μm. **E** Percentage of atherosclerotic lesions in the whole cross-sectional arterial area was quantified (*n* = 7). Data are expressed as median with interquartile range (**B**) and mean ± SEM (**E**). Statistics: Kruskal–Wallis test with Dunn ‘s correction for 2 comparisons in (**B**); Unpaired t test in (**E**). * *P* < 0.05, ***P < 0.001, ****P < 0.0001

To determine if 12/15-LOX mediates disturbed flow-driven atherogenesis, ApoE^−/−^ and ApoE^−/−^/ALOX15 ^−/−^ mice fed with HFD were subjected to partial ligation of left carotid arteries. After 2 weeks, ORO staining demonstrated ~ eightfold increase in atherosclerotic lesion areas proximal to the ligation site of left carotid artery compared to the non-operated contralateral side (right carotid artery) in ApoE^−/−^ mice. Of note, areas of atherosclerosis were largely decreased by 12/15-LOX deficiency (Fig. 6A and B). Similarly, lesion area was significantly decreased in partially-ligated carotid arteries of ApoE^−/−^ mice receiving intraperitoneal injection of baicalein compared to the control group (Fig. 6A and B).

To confirm the role of endothelial 12/15-LOX in the development of atherosclerosis in disturbed flow areas, 12/15-LOX expression in endothelium was specifically knocked-down by intravenous injection of AAV-mediated delivery of ALOX15 -shRNA under the EC-specific Cdh5 promoter (Fig. 6C and Supplementary Fig. 8A). We found that EC-specific 12/15-LOX knockdown markedly reduced atherosclerosis in ApoE^−/−^ mice after carotid artery partial ligation (Fig. 6D and E).

## Discussion

In this study, we demonstrated that endothelial 12/15-LOX is a critical player in disturbed flow-induced atherosclerosis. First, we showed that 12/15-LOX is a mechano-sensitive factor significantly induced by disturbed flow in ECs. Second, endothelial 12/15-LOX plays a pro-atherogenic role through multiple mechanisms including catalyzing the production of eicosanoid metabolites and promoting EC-leukocyte interactions. Third, transactivation of the ALOX15 promoter by a mechanosensitive transcription factor SREBP2 is responsible for its upregulation under disturbed flow. Finally, pharmacological inhibition or genetic deletion of 12/15-LOX significantly attenuated the development of atherosclerosis, which provides a molecule target to treat atherosclerosis especially at disturbed flow sites.

Disturbed flow profoundly upregulates adhesion molecules on ECs, facilitating the recruitment of circulating leukocytes to the vessel wall and triggering a cascade of inflammatory responses that promote atherosclerosis progression (Brown, et al. 2016). Previous studies showed that 12/15-LOX promoted cell adhesion molecule expression and endothelial tight junction disruption through differential signaling pathways (Bolick, et al. 2005, Bolick, et al. 2006, Kundumani-Sridharan, et al. 2013). In this study, we showed that disturbed flow activated mechano-sensitive SREBP2 to transcriptionally upregulate 12/15-LOX, which directly drives EC dysfunction. We found that the knockdown of SREBP2 resulted in reduced expression levels of 12/15-LOX under disturbed flow, with no significant difference compared to steady flow. Knockdown of SREBP2 led to reduced expression of 12/15-LOX under disturbed flow while had no effects under steady flow (*P* = 0.711), indicating that the SREBP2-12/15-LOX mechanism driven by disturbed flow may not generally apply to other hemodynamic conditions. However, whether other mechanosensitive transcription factors, such as KLF2 or KLF4 (Fan, et al. 2017, Sweet, et al. 2018) modulate the regulation of 12/15-LOX under different patterns of shear stress awaits further investigation. On the other hand, we found that the overexpression of precursor SREBP2 in HUVECs under steady flow did not enhance ALOX15 promoter activity. In contrast, overexpressing precursor SREBP2 in HUVECs exposed to disturbed flow markedly transactivated the ALOX15 promoter. A previous report showed that the overexpression of the mature form of SREBP2 alone was sufficient to induce endothelial inflammation (Xiao, et al. 2013). These findings support the notion that disturbed flow may sustain SREBP activation, while steady flow only triggers transient activation (Liu, et al. 2002, Zeng, et al. 2004), further explaining the difference in SREBP2 maturation and ALOX15 transactivation under different shear stress patterns.

Our in vivo and in vitro studies clearly showed that 12/15-LOX depletion attenuated VCAM-1 and ICAM-1 upregulation induced by disturbed flow. Furthermore, EC-monocyte interactions were profoundly decreased in ALOX15 knockout conditions compared to controls especially under disturbed flow exposure. Interestingly, Huo et al. reported that expression of VCAM-1 and ICAM-1 did not significantly differ between wild-type and 12/15-LOX-deficient aortic ECs either under resting conditions or activated by TNF, or LDL (Huo, et al. 2004). A plausible explanation for this discrepancy is that 12/15-LOX-mediated EC adhesion molecule expression might be more significant when activated by mechanical stress than these biochemical factors, which awaits validation in the further studies. Mechanistically, 12/15-LOX-dependent metabolites have been identified as essential mediators of ECs inflammation. For example, 12/15-LOX overexpression promotes ICAM-1 expression and monocyte adhesion to endothelium via its PUFA metabolites such as 12(s)-HETE through NF-$\kappa$ B and RhoA pathway (Bolick, et al. 2005, Bolick, et al. 2006, Reilly, et al. 2004). In pulmonary vasculature, 15 s-HETE exacerbates pulmonary hypertension by activating ECs-leukocytes interactions (Ruffenach, et al. 2020). 15 s-HETE also induced aortic ring contraction through activating TXA₂ receptor (Tesfamariam, et al. 1995) and suppressing NO production (Chawengsub, et al. 2009). In ECs under disturbed flow, we found a 12/15-LOX-dependent increase in 15 s-HETE, but not 9 s-/13 s-HODE or 12 s-HETE. The upregulation of 15 s-HETE was abolished by 12/15-LOX deficiency or pharmacological inhibition without compensatory changes in other arachidonic acid-derived products (e.g., COX, cP450). Supplementation of 15 s-HETE reverted the 12/15-LOX-deficient ECs to a dysfunctional phenotype under disturbed flow. LOX, COX, and CYP450 are critical enzymes that play pivotal roles in the metabolism of arachidonic acid. A previous study showed that COX-2 is regulated by fluid shear stress at both transcriptional and post-transcriptional levels (Russell-Puleri, et al. 2017). In this study, we found that disturbed flow significantly upregulated the levels of 6-keto-PGF1α, PGE2 and PGF2α, which are all converted from PGH2, a major product of arachidonic acid when catalyzed by COX-1 or COX-2. Accordingly, knockdown of ALOX15 did not reverse the production of these COX-derived eicosanoid metabolites. A number of isoforms of CYP450 have been confirmed to be regulated by shear stress (Ghosh, et al. 2010). Certain CYP450 enzymes can also metabolize arachidonic acid to produce 12 s-/15 s-HETE or metabolize linoleic acid to from 9 s-/13 s-HODE (Wang, et al. 2021). Furthermore, these PUFA metabolites can be further oxidized by CYP450 enzymes or various dehydrogenases, leading to the production of a complex mixture of oxygenated products with altered biological activities (Kirpich, et al. 2016, Powell, et al. 2015). Among them, 15 s-HETE appears to be predominantly catalyzed by 12/15-LOX in response to disturbed flow. However, supplementation of 9 s-/13 s-HODE and 12 s-HETE can also restore the responsiveness of 12/15-LOX-deficient ECs to disturbed flow. Precise characterization of the expression and activities of these enzymes including 12/15-LOX in response to hemodynamic shear stress warrants further studies and is our future goal.

Furthermore, multiple vascular cell types are activated and promote disease development during the pathogenies of atherosclerosis, such as VSMC migration and phenotypic switching, and macrophage infiltration. These processes are also under regulation by 12 s-HETE (Kriska, et al. 2022, Yuyu Li 2024) and 15 s-HETE (Singh, et al. 2011) via certain pathways, implying that hemodynamic shear stress may initiate the pro-atherogenic cascade via 12/15-LOX-derived metabolites in ECs which subsequently affect other vascular cells.

By performing partial carotid ligation in ApoE^−/−^ and ApoE^−/−^/ALOX15^−/−^ mice, we showed that atherosclerosis under disturbed flow was attenuated by 12/15-LOX deficiency. Plasma lipid profiles were measure in ApoE^−/−^ mice, ALOX15-/-/ApoE^−/−^ mice, and baicalein-treated ApoE^−/−^ mice, with no statistically significant differences between groups (Cyrus, et al. 2001, Cyrus, et al. 1999, George, et al. 2001, Li, et al. 2018). Indeed, 12/15-LOX has a wide tissue distribution. The role of 12/15-LOX in macrophages during atherogenesis has been well-recognized, predominantly through promoting lipid peroxidation, cytokine production, and foam cell formation (Huo, et al. 2004). Moreover, reduced growth factor-induced migration, proliferation, fibronectin production by 12/15-LOX deficiency in vascular smooth muscle cells have also been identified (Reddy, et al. 2003). To further confirm the role of endothelial 12/15-LOX, we established EC-specific 12/15-LOX knockdown mice through AAV-shRNA controlled by an EC-specific Cdh5 promoter, which also exhibited a significant alleviation of disturbed flow-induced atherosclerosis after EC-specific 12/15-LOX knockdown. These data jointly support the notion that 12/15-LOX widely participates the development of atherosclerosis especially under certain pro-atherogenic stimuli such as disturbed flow and may behave differentially in different vascular cell types under different pathophysiological conditions.

In a translational perceptive, we used a 12/15-LOX inhibitor baicalein to inhibit 12/15-LOX activity. We demonstrated that baicalein reversed disturbed flow-induced endothelial dysfunction and the progression of atherosclerosis, with a similar trend as observed in ALOX15 knockout mice. In PUFA metabolite analysis, we found that both 12/15-LOX-dependent metabolites were decreased in ECs treated with baicalein. More importantly, as the safety of baicalein tablets has been evaluated in phase IIa clinical trial in healthy adults with influenza fever (clinical trials registration numbers NCT03830684), our findings provide therapeutic potentials of baicalein in atherosclerotic cardiovascular disease.

In conclusion, our study uncovers a novel mechanism by which disturbed flow drives endothelial dysfunction through promoting 12/15-LOX via a SREBP2-dependent pathway, which orchestrates the production of pro-inflammatory PUFA metabolites. By demonstrating that 12/15-LOX serves as a critical link between hemodynamic forces and endothelial dysfunction, we provide novel insights into the molecular basis of flow-induced vascular pathology. These findings not only advance the understanding of the role of 12/15-LOX in atherosclerosis but also highlight the SREBP2-12/15-LOX axis as a promising therapeutic target for mitigating endothelial dysfunction and atherosclerosis under disturbed flow.

## Supplementary Information

Supplementary Material 1.

## Data Availability

No datasets were generated or analysed during the current study.

## Abbreviations

EC: Endothelial cell
ICAM-1: Intercellular adhesion molecule 1
PUFA: Polyunsaturated fatty acids
VCAM-1: Vascular cell adhesion molecule 1
LC–MS/MS: Liquid chromatography-mass spectrometry
H&E: Hematoxylin and eosin
ORO: Oil red O
AAV: Adeno-associated virus
Cdh5: VE-cadherin
ApoE: Apolipoprotein E
