# Supplementary material for: 12/15-lipoxygenase mediates disturbed flow-induced endothelial dysfunction and atherosclerosis
Source: Mol Med. 2025 Jul 15;31:257. doi: 10.1186/s10020-025-01297-0 (PMC12261642; doi:10.1186/s10020-025-01297-0)
Supplement: Supplementary file 1 — Supplementary Material 1. [file 10020_2025_1297_MOESM1_ESM.pdf]

Supplementary Materials

12/15-Lipoxygenase Mediates Disturbed Flow-induced Endothelial Dysfunction and Atherosclerosis

Jia Wei, Chen[1,2#]; Shi Li, Chen[3#]; Xin Rui, Wu[1,2#]; Xin Yi, Shu[1,2]; Si Yi, Tang[1,2]; He, Yuan; You Ran, Li; Jin Wei, Quan[1,2]; Shuo Feng[1]; Feng Hua, Ding[1]; Rui Yan, Zhang[1,2]; Wei Feng, Shen[1,2]; Chen Die, Yang[1,2*]; Lin Lu[1,2*]; Xiao Qun, Wang[1,2*]

# These authors contributed equally to this work

*Corresponding authors:

Xiao Qun Wang, M.D., Ph.D., E-mail: wangxq@shsmu.edu.cn; Xiaoqun_Wang@hotmail.com

Lin Lu, M.D., Ph.D., E-mail: rjlulin1965@163.com

Chen Die Yang, M.D., Ph.D., E-mail: yangcd@shsmu.edu.cn

Department of Cardiovascular Medicine, Rui Jin Hospital

197 Ruijin Er Rd

Shanghai, 200025

China

This file includes:

Supplementary Figure. 1 to Supplementary Figure. 6

Supplementary Methods

Figure S1

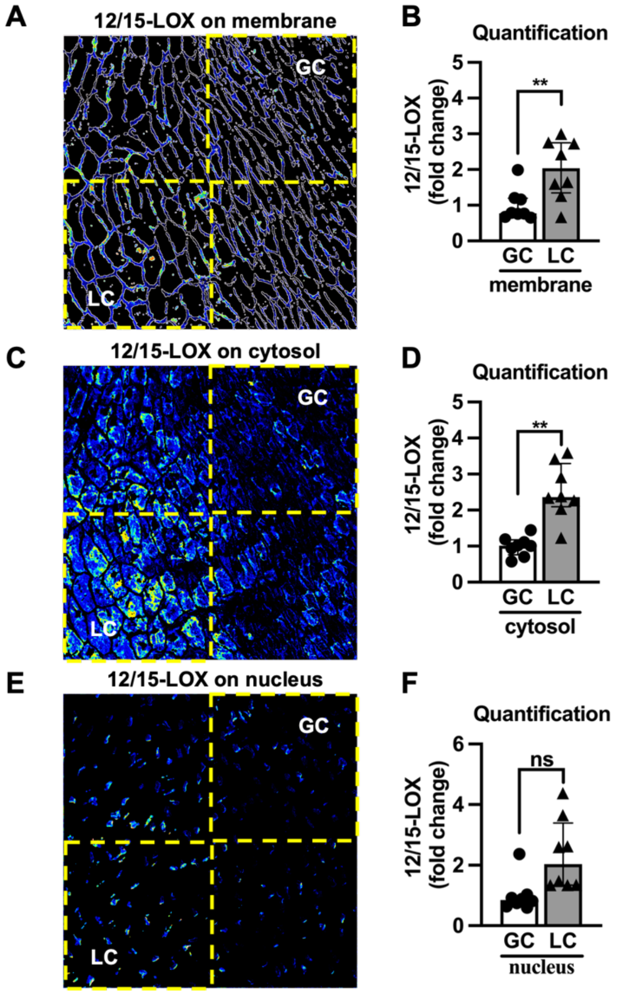

Supplemental Figure S1. Quantification of 12/15-LOX subcellular expression

Representative reconstructed heatmaps and quantification of immunofluorescence staining of 12/15-LOX expression in **(A-B)** membrane, **(C-D)** cytosol and **(E-F)** nucleus of aortic endothelial cells in mice (n=8). Cells membranes were outlined by VE-cadherin and nuclei were counterstained with DAPI.

Data are expressed as median with interquartile range (B, D, F). Wilcoxon test for comparisons of 12/15-LOX expression in membrane, cytosol and nucleus of GC and LC in B, D, F. **P<0.01. ns, no significant difference.

Figure S2

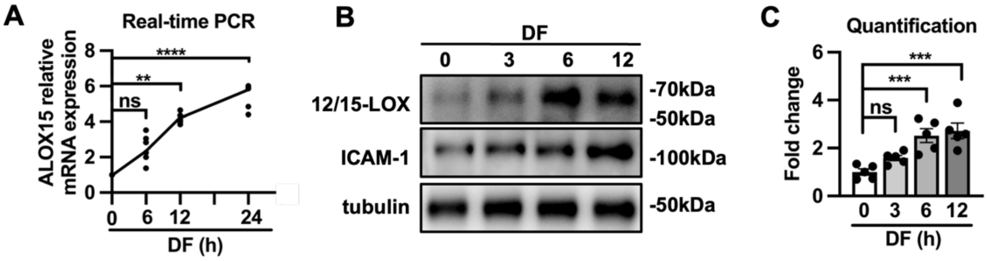

Supplemental Figure S2. 12/15-LOX were increased in response to disturbed flow in a time-dependent manner.

(A) Real-time PCR were performed to quantify ALOX15 mRNA levels in HUVECs exposed to disturbed flow (DF) at indicated time points (n=6).

(**B-C**) HUVECs were exposed to disturbed flow at indicated time points. Protein levels of 12/15-LOX, ICAM-1 were analyzed by Western Blot and (**C**) quantified (n=5).

Data are expressed as the mean ± SEM (C). Statistics: Kruskal-Wallis test with Dunn‘s correction for 2 comparisons in A; 1-way ANOVA with Holm-Šídák's post hoc test for 3 comparisons in C. **P<0.01, ***P<0.001, ****P<0.0001. ns, no significant difference.

Figure S3

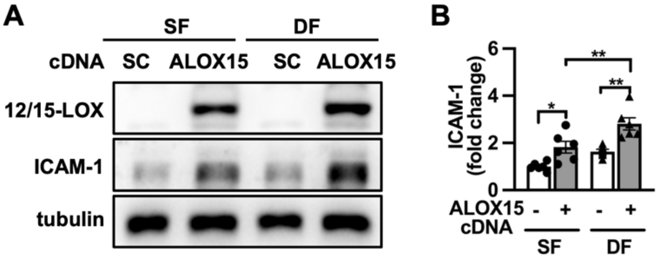

Supplemental Figure S3. Overexpression of 12/15-LOX promotes disturbed flow-elicited EC inflammation

(A) HUVECs transduced with lentivirus expressing scramble (SC) or ALOX15 cDNA were exposed to either steady flow or disturbed flow for 24 hours. Protein levels of 12/15-LOX, ICAM-1 were analyzed by Western Blot and (**B**) quantified (n=6).

Data are expressed as the mean ± SEM (B). 2-way ANOVA with Holm-Šídák's post hoc test for 3 comparisons in B. * $P < 0.05$, **$P<0.01$.

Figure S4

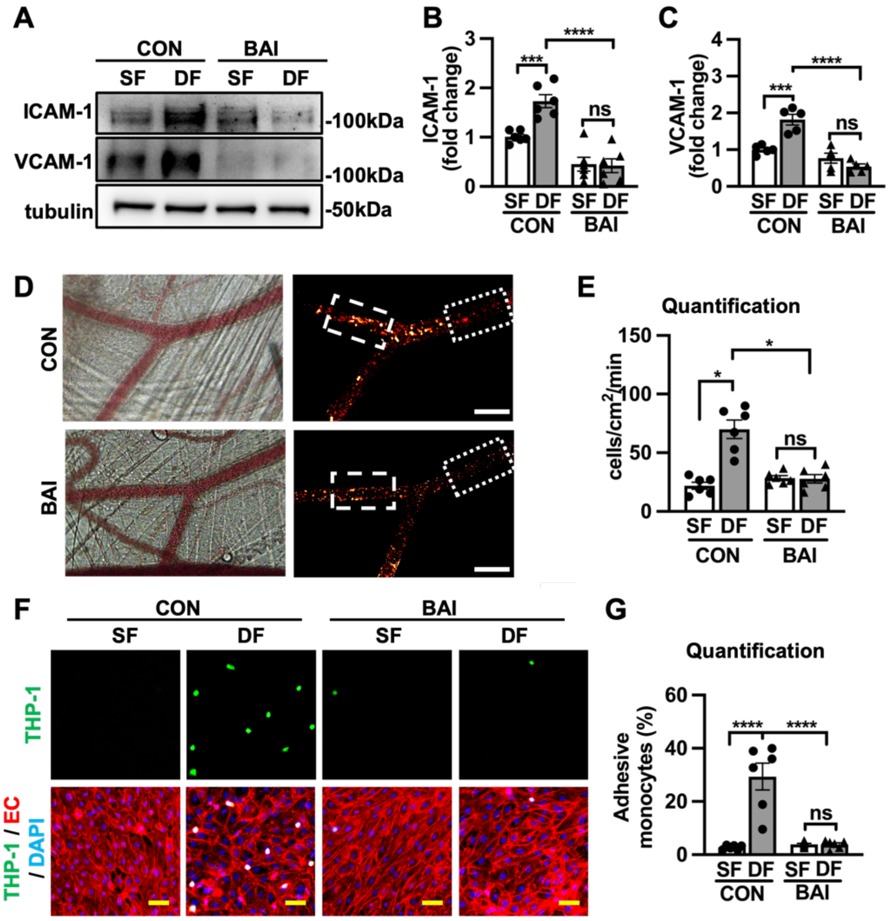

Supplemental Figure S4. Inhibition of 12/15-LOX activity attenuates disturbed flow-elicited EC inflammation

(A-C) HUVECs pretreated with baicalein (10µM) or vehicle for 2 hours were exposed to steady flow or disturbed flow for 24 hours. Protein levels of ICAM-1 **(B)** and VCAM-1 **(C)** were analyzed by Western Blot and quantified (n=5-6).

(D-E) Representative heatmaps show overlaying images over a 10 second period (57 frames/second) of leukocytes marked by Rhodamine 6G staining in bifurcated arteries of cremaster muscle in ApoE$^{-/-}$ mice injected with baicalein (100mg/kg) or vehicle. Dotted rectangles indicate arterial steady flow regions, dashed rectangles indicate arterial disturbed flow region. Scale bar, 100µm. **(E)** Bar graph shows the number of rolling leukocytes (n=6).

(F-G) HUVECs pretreated with baicalein (10µM) or vehicle were exposed to steady flow or disturbed flow, followed by coincubation with THP-1 monocytes labeled by Calcein-AM for 1 hours. Adhesive THP-1 monocytes remained after PBS rinse and **(G)** quantified as percentage of THP-1 monocytes to HUVECs (n=6). Scale bar, 50µm.

Data are expressed as the mean ± SEM (B, C, E and G). 2-way ANOVA with Holm-Šídák's post hoc test for 3 comparisons in B, C and G. Repeated measures ANOVA with Holm-Šídák's post hoc test for 3 comparisons in E. * $P < 0.05$, ***$P<0.001$, ****$P<0.0001$. ns, no significant difference.

Figure S5

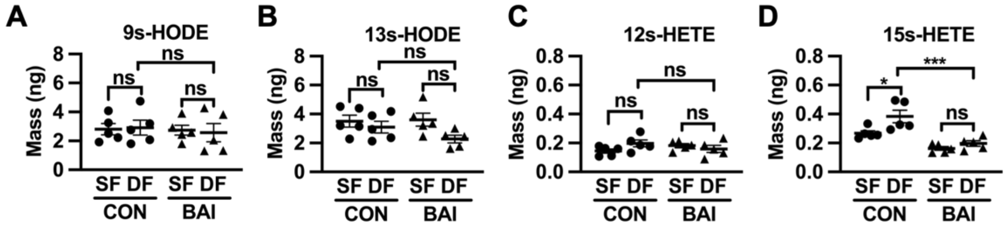

Supplemental Figure S5. 12/15-LOX inhibition attenuates disturbed flow-Induced upregulation of 15s-HETE in ECs.

(**A-D**) HUVECs were pretreated with baicalein (10μM), followed by exposure to steady flow or disturbed flow for 24 hours. 9s-HODE, 13s-HODE, 12s-HETE and 15s-HETE levels (ng/5×10^6 cells) in HUVECs were determined by LC-MS/MS analysis (n=5).

Data are expressed as the mean ± SEM (A through D). Statistics: 2-way ANOVA with Holm-Šídák's post hoc test for 3 comparisons in A through D. * $P < 0.05$, ***$P<0.001$. ns, no significant difference.

Figure S6

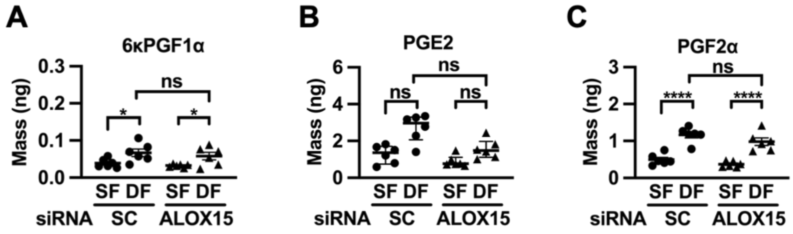

Supplemental Figure S6. Effects of 12/15-LOX on cyclooxygenase (COX) PUFA metabolites levels in ECs under disturbed flow

(**A-C**) HUVECs transfected with ALOX15 siRNA were exposed to steady flow or disturbed flow for 24 hours.

(**A**) 6κPGF1α, (**B**) PGE2, (**C**) PGF2α levels (ng /5×10^6 cells) in HUVECs were determined by LC-MS/MS analysis (n=6).

Data are expressed as the mean ± SEM (A and C); median with interquartile range (B). 2-way ANOVA with Holm-Šídák's post hoc test for 3 comparisons in A and C; Kruskal-Wallis test with Dunn's correction for 3 comparisons in B. * $P < 0.05$, ****$P<0.0001$. ns, no significant difference.

Figure S7

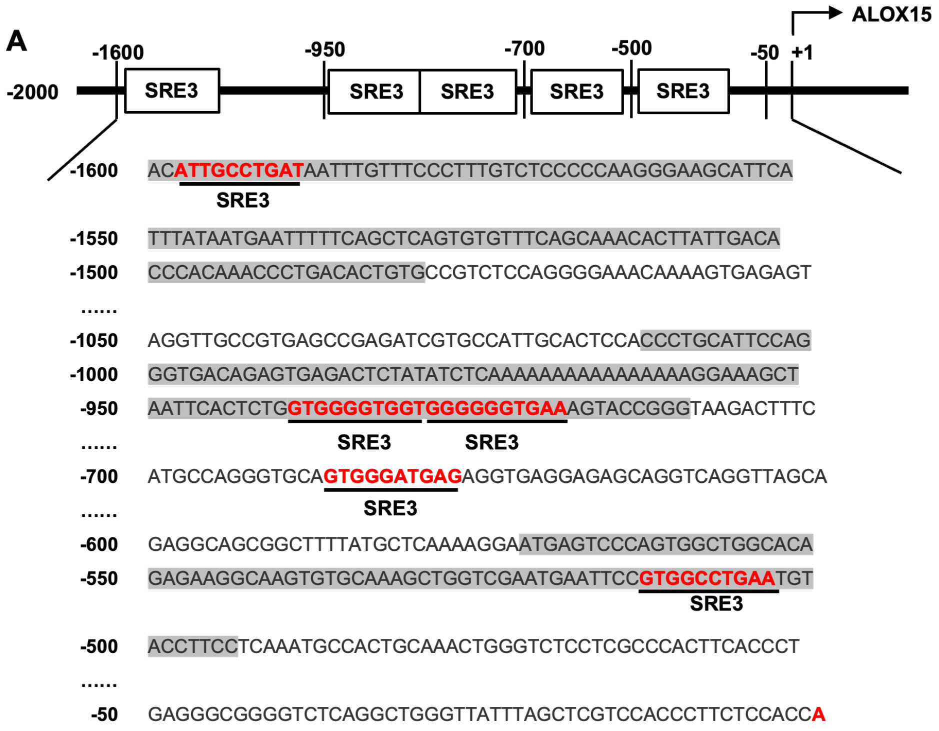

Supplemental Figure S7. Analysis of SREBP2 binding sites on ALOX15 promoter

(A) ALOX15 promoter sequence. SREBP2 binding sites are indicated in red font and shown below the sequence. The sequences validated by ChIP-qPCR with statistical significance are highlighted.

Figure S8

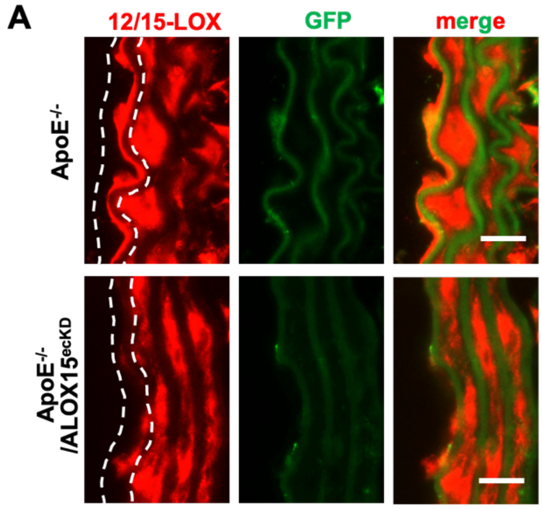

Supplemental Figure S8. Validation of endothelial-specific ALOX15 knockdown in mice using AAV-mediated shRNA delivery

Representative images of immunofluorescence staining of 12/15-LOX or GFP in arteries from mice intravenously injected with AAV- ALOX15 shRNA or control virus. Dashed area indicates endarterium. Scale bar, 20μm.

Materials and reagents

**Animals (in vivo studies)**				
Species	Vendor	Background Strain	Sex	Catalog #
Wildtype mouse	Charles River	C57BL/6J	Male and female	219

**Genetically Modified Animals**				
	Species	Vendor	Background Strain	Catalog #
ALOX15$^{-/-}$ Parent - Male	Mouse	Jackson Lab	C57BL/6J	002778
ALOX15$^{-/-}$ Parent- Female	Mouse	Jackson Lab	C57BL/6J	002778
ApoE$^{-/-}$ Parent - Male	Mouse	Charles River	C57BL/6J	
ApoE$^{-/-}$ Parent - Female	Mouse	Charles River	C57BL/6J	

**Antibodies**			
Target antigen	Vendor	Catalog #	Working concentration
CD31	SIGMA-ALDRICH	p8590	1:100 for IF
12/15-LOX	Abcam	ab80221	1:100 for IF
12/15-LOX	SANTA CRUZ	sc-133085	1:500 for WB
VE-Cadherin	BD-Pharmingen	555289	1:100 for IF
ICAM-1	CST	4915S	1:1000 for WB
VCAM-1	CST	13662S	1:1000 for WB
VCAM-1	Abcam	ab134047	1:100 for IF
Tubulin	Beyotime	AT819	1:1000 for WB
SREBP2	Cayman	10007663	1:1000 for WB
SREBP2	BD-Pharmingen	557037	1:50 for ChIP
normal rabbit IgG	SANTA CRUZ	sc-2027	1:200 for IF
Anti-mouse IgG, HRP-linked Antibody	CST	7076	1:3000 for WB
Anti-rabbit IgG, HRP-linked Antibody	CST	7074	1:3000 for WB
Donkey anti-Rat, Alexa Fluor™ 555	Invitrogen	A-78945	1:2000 for IF
Donkey anti-Rabbit, Alexa Fluor™ 488	Invitrogen	A-21206	1:2000 for IF
Donkey anti-Mouse, Alexa Fluor™ 555	Invitrogen	A-31570	1:2000 for IF

**DNA/cDNA Clones**		
Clone Name	Vendor	Catalog #
2 × FLAG-SREBP-2	Addgene	26807
pGL4.10-ALOX15-promoter-wt (-1/-2000bp)	this manuscript	
pGL4.10- ALOX15-promoter-wt (-1/-1400bp)	this manuscript	
pGL4.10- ALOX15-promoter-wt (-1/-800bp)	this manuscript	
pGL4.10- ALOX15-promoter-wt (-1/-200bp)	this manuscript	

**Virus**	

Clone Name	Vendor
pcSLenti-EF1-EGFP-P2A-Puro-CMV-MCS-3×FLAG-WPRE	this manuscript
pcSLenti-EF1-EGFP-P2A-Puro-CMV-ALOX15-3×FLAG-WPRE	this manuscript
pAAV-Cdh5 Promoter-EGFP-3xFLAG-miR30 shRNA (NC)-tWPA	this manuscript
pAAV-Cdh5 Promoter-EGFP-3xFLAG-miR30 shRNA (ALOX15)-tWPA	this manuscript

Cultured Cells

Name	Vendor	Sex (F, M, or unknown)	Catalog #
HUVECs	Promocell	unknown	C-12203
THP-1	Human monocyte cell line	unknown	
293T	Human epithelial-like cell line	unknown	

Primers

Name of Target	Forward Primer (5’ to 3’)	Reverse Primer (5’ to 3’)
Mouse ALOX15	CGCCTGCAGCCAGACATGGTA	GGCAGGTCAAGTTGCTCATTCCG
Mouse GAPDH	TGGCAAAGTGGAGATTGTTGCC	AAGATGGTGATGGGCTTCCCG
Human ALOX15	GCATGCATGCATGCATGCAT	CAGTCAGTCAGTCAGTCAGT
Human GAPDH	GGAGCGAGATCCCTCCAAAAT	GGCTGTTGTCATACTTCTCATGG
ChIP-qPCR amplicon		
VI (-1624 to -1521)	CCCCTTTTGTTTACATTGCCTG	AGTGTTTGCTGAAACACACTGAGC
V (-1579 to -1488)	CCCAAGGGAAGCATTCATTTATAATG	ACGGCACAGTGTCAGGGTTTGT
IV (-1062 to -960)	GGTGACAGAGTGAGACTCTATATCTC	CAGGAAAGTCTTACCCGGTAC
III (-998 to -955)	GGGTGGTGGGGGGTGAAAG	TTTCTCAGGAAAGTCTTACCCGGTAC
II (-702 to -589)	GCAGTGGGATGAGAGGTGAGGAG	TTTGAGCATAAAAGCCGCTGC
I (-571 to -493)	GCTGGCACAGAGAAGGCAAG	CAGTGGCATTTGAGGAAGGTAC

Small interfering RNA (siRNA)

ALOX15	GUCGAGAGAUCACUGAAAUTT	AUUUCAGUGAUCUCUCGACTT
SREBP2	GCUGGUUUGACUGGAUGAUTT	AUCAUCCAGUCAAACCAGCTT
scramble	UUCUCCGAACGUGUCACGUTT	ACGUGACACGUUCGGAGAATT

Others

Description	Vendor	Catalog #
Magna ChIP HiSens Chromatin IP Kit	Merck	MAGNA0025
Promega Dual-Luciferase™ Reporter (DLR™) Assay Systems	Promega	E1910
FastQuant RT Kit (With gDNase)	Tiangen	KR106
Vybrant® Cell Adhesion Assay Kit	Thermo Fisher	V13181
Rhodamine 6G	sigma	83697
Hieff® qPCR SYBR Green Master Mix (High	YEASEN	11203ES08

Rox Plus)		
Immobilon Western Chemiluminescent HRP Substrate	Millipore	WBKLS0500
DAPI	Roche	10236276001
Hoechst 33342 Solution	Thermo Fisher	62249
Alexa Fluor™ 555 Phalloidin	Thermo Fisher	A34055
Baicalein	Selleckchem	S2268
PMSF	Beyotime	ST506
Protease Inhibitors cocktail	Bimake	B14001
RIPA lysis buffer	Beyotime	P0013B
Endothelial Cell Medium	Sciencell	SC-1001
RPMI	thermofisher	22400089
Opti-MEM I	thermofisher	31985070
lipofectemine 2000	Invitrogen	11668019
Lipofectamine® RNAiMAX	Invitrogen	13778150
O.C.T. Compound	Sakura	4583
TRIZOL REAGENT	thermofisher	15596018
Hematoxylin	Baso	BA-4041
Eosin	Baso	BA-4022
Oil Red O	SIGMA ALDRICH	O0625-25G
5(S)-HETE-d8	Cayman chemical	334230
12(S)-HETE-d8	Cayman chemical	334570
15(S)-HETE-d8	Cayman chemical	334720
13(S)-HODE-d4	Cayman chemical	338610
9(S)-HODE-d4	Cayman chemical	338410
6k PGF1α-d4	Cayman chemical	315210
PGE2-d4	Cayman chemical	314010
11,12 EET-d8	Cayman chemical	10006413
9s-HODE	SIGMA ALDRICH	SML0503-100UG
13s-HODE	APExBio	C3604
12s-HETE	Cayman chemical	34570
15s-HETE	APExBio	C4187

Human arterial specimens

Human internal mammary arteries were obtained from patients undergoing coronary artery bypass grafting, with informed consent and approval from the Ethics Committee of Ruijin Hospital, Shanghai Jiao-Tong University School of Medicine. Inclusion criteria: (1) age ≥18 and ≤85 years; (2) patients diagnosed with coronary artery disease (CAD) undergoing coronary artery bypass grafting. We controlled for systematic factors which may influence 12/15-LOX expression (Chawengsub, et al. 2009, McDuffie, et al. 2008) by excluding patients with: (1) history of

myocardial infarction; (2) diabetes mellitus; and (3) poorly controlled blood pressure (systolic blood pressure >140 mmHg or <90 mmHg). The patient information is provided in the following list.

	**Gender**	**Age**	**Stage of Disease**	**Tissue**
Sample 1	male	61	SYNTAX III	Left intramammary artery
Sample 2	male	53	SYNTAX III	Left intramammary artery
Sample 3	male	76	SYNTAX III	Left intramammary artery
Sample 4	male	71	SYNTAX III	Left intramammary artery
Sample 5	male	78	SYNTAX III	Left intramammary artery
Sample 6	male	47	SYNTAX III	Left intramammary artery

Animals

Mice with global Alox15 gene deletion (C57BL/6 background) were purchased from The Jackson Laboratory (Bar Harbor, Marine, USA). C57BL/6 and $ApoE^{-/-}$ mice were purchased from Charles River Laboratory Animal Technology Co., Ltd (Beijing, China). Then $ALOX15^{-/-}$ mice were crossed with $ApoE^{-/-}$ mice to generate $ApoE^{-/-}$ / $ALOX15^{-/-}$ mice, as previously described(Li, et al. 2018). Control mice with the same age and sex from littermates were used in experiments involving in $ALOX15^{-/-}$ mice. To evaluate the potential role of 12/15-LOX in the vascular endothelium of mice, we established AAV-mediated delivery systems expressing either ALOX15 specific shRNA or scramble control shRNA, both driven by the endothelial cell-specific Cdh5 promoter. The constructs, pAAV-Cdh5-EGFP-3xFLAG-miR30 shRNA (ALOX15)-tWPA and its control virus, were packaged into AAV particles for in vivo delivery. Virus (5×10^{11} gc/mice) were delivered to $ApoE^{-/-}$mice (male, 8 weeks old) via tail vein injection. 3 weeks after injection, the efficiency of virous was evaluated and mice were subjected to carotid artery partial ligation or sham surgery and high fat diet for 2 weeks. To examine the pharmacological and

therapeutic effects of baicalein, $ApoE^{-/-}$ mice were intraperitoneally injected with baicalein (20mg/kg/day) 2 days before partial carotid ligation and continued until euthanasia. To establish animal models, mice anesthesia was induced by inhalation of 3-5% isoflurane and then maintained by 1-2% isoflurane with a small rodent respirator during surgery. For euthanatizing, mice were anesthetized with intraperitoneal injection of sodium pentobarbital (50 mg/kg) and heparin and euthanatized by cervical dislocation. The mice were acclimated to the laboratory environment for one week before experimentation.

All animal procedures were performed in accordance with the National Institutes of Health (NIH) and approved by Ethics Committee of Ruijin Hospital, Shanghai Jiao-Tong University School of Medicine.

Partial ligation surgery and vascular ultrasonography

LCA partial ligation surgery were performed as described in reference(Nam, et al. 2009). Briefly, after mice anesthesia induced as mentioned above, a midline ventral incision (8-10 mm) was made in neck epilated area. Muscles in carotid triangles were bluntly dissected to expose LCA (left common carotid artery) and RCA (right common carotid artery). Three distal branches of LCA (occipital artery, internal carotid and left external carotid artery) were tightly ligated by 6-0 silk suture, with left superior thyroid artery remaining intact. After ligation, the incision was sutured with 3-0 silk suture. Measures were taken to maintain mice body temperature, pain relief, and infectious prevention. C57BL/6 mice was fed with chow diet for 2 weeks post-operation. Mice with $ApoE^{-/-}$ background were fed with high fat diet (40 kcal% fat, 1.25% cholesterol, 0.5% cholic acid, cat# D12109, Research Diets, Inc., New Brunswick, NJ) from the day after surgery to 2 weeks. Echography was performed before surgery, 1 day and 2 weeks after surgery to measure common carotid arteries flow velocity in pulse wave doppler mode. All ultrasound measurements were taken in anesthetized mice with Vevo 2100 high-resolution micro-ultrasound imaging system (Toronto, Canada).

Intravital microscopy

Mice were anesthetized as mentioned above. Cremaster muscle was isolated, and arterioles were observed by microscope. Rolling and adhesive leukocytes labeled by 0.4mg/ml Rhodamine 6G via retro-orbital injection were then monitored and recorded. Fiji imageJ were used to analyze the numbers of rolling and adhesive leukocytes in arterioles of certain regions in a certain period.

Cell culture and application of shear stress in vitro

HUVECs were cultured in endothelial cell medium (ECM) with 5% fetal bovine serum (FBS), 100 μg/ml streptomycin and 100 IU/ml penicillin in humidified cell culture incubator (37°C, 95% air and 5% CO_2). THP-1 were cultured in RPMI medium with 10% FBS, 100 μg/ml streptomycin and 100 IU/ml penicillin in the same environment as HUVECs.

IBIDI flow pump system and an orbital shaker were used to generate steady flow or disturbed flow on HUVECs as described(Liu, et al. 2017). HUVECs were seeded on IBIDI μ-slides coated with adhesion factor. The slide was connected to the IBIDI perfusion set when cells at 100% confluence. For steady flow, HUVECs were exposed to shear stress of 13 dyn/cm^2. For disturbed flow, cells were exposed to shear stress of ±5 dyn/cm^2. To collect a large amount of cells for biochemical analysis, HUVECs plated on 6-well plate were set up into an orbital shaker that sheared at 180 rpm (~14 dyn/cm^2) for steady flow or at ±90 rpm (±5 dyn/cm^2) for disturbed flow. The shear stress imposed on the HUVEC was calculated by the formula $\alpha\sqrt{\eta\rho(2\pi f)^3}$ where α is the radium of rotation, η is the viscosity of medium, ρ is the density of medium, f is the rotation speed. To generate steady flow by the orbital shaker, cells were segmented by a patch of Pluronic F-127 solution as described by Fernandes (Fernandes, et al. 2022) and Ghim (Ghim, et al. 2018) with modification. Briefly, to prevent cells growth in the area of disturbed flow, 6-well plates were coated with 1% Pluronic F-127 solution from the center to radius of 7.84 mm in the bottom of the wells. After one hour coating and rinse with PBS, HUVECs were plated and grew to confluence only in the area not coated with the 1% Pluronic F-127 solution. Afterwards, we harvested cells in the outer area under exposure to steady flow. To keep consistency, cells treated with disturbed flow were also cultured in such pretreated plates and thus only grew in the outer area.

Immunofluorescence

Human internal mammary arteries were embedded in optimal cutting temperature (OCT) compound in -80°C and sectioned at a thickness of 6μm, followed by permeabilizing and blocking with 10% normal goat serum in PBS containing 0.2% Triton-100 for 1 hours at room temperature. The sections were incubated with primary antibodies against CD31, 12/15-LOX or normal rabbit IgG diluted in PBS containing 0.1% Triton-100 and 2% normal goat serum. After incubation at 4°C overnight, sections were stained with fluorescence-conjugated secondary antibodies for 2 hours. Nuclei were counterstained with 4',6-diamidino-2-phenylindole (DAPI) for 10 minutes.

En face immunofluorescence staining of mouse aorta was performed as described previously(Li, et al. 2019, Wang, et al. 2012). Briefly, 12-week-old C57BL/6 mice were anesthetized as mentioned above. After cutting off the jugular vein, pressure perfusion (100mmHg) was performed with pre-chilled saline containing 40U/ml heparin for 5 minutes from the mice left ventricular apex, followed by perfusion with pre-chilled 4%(wt/vol) paraformaldehyde for 5 minutes. Then the aorta with arterial tree, including left subclavian artery, left common carotid artery and innominate artery, were dissected from heart to celiac trunk and cut open longitudinally along the lesser curve. After permeabilization and blocking as mentioned above, the aorta was incubated with anti-12/15-LOX antibody and anti-CD144 antibody overnight at 4°C, followed by fluorescence-conjugated secondary antibodies incubation. Nuclei were counterstained with DAPI for 10 minutes.

Staining was visualized with Olympus FV3000 or Olympus IX81 confocal microscope. In each experiment, images of the same staining maker were taken under consistent conditions. 12/15-LOX fluorescence intensity in indicated regions was quantified using Fiji ImageJ. In Figure 1, CD31-positive areas were thresholded and converted to ROI (Region of Interest). In Figure 2 and Figure 3, great curvature or lesser curvature and steady flow region near renal artery ostium or disturbed flow region of renal artery ostium based on anatomical structures were defined as ROI. 12/15-LOX fluorescence integrated density within these ROIs was measured after background subtraction.

Histology

For Hematoxylin and eosin staining, sections were stained with Hematoxylin for 3 minutes and eosin for 5 minutes. For Oil Red O (ORO) staining, sections were fixed with 4%(wt/vol) paraformaldehyde and then stained with filtered ORO solution (2.5mg/ml) for 30 minutes and then rinsed with 60% isopropanol for 10 seconds. Nuclei were counterstained with hematoxylin before mounting. Images were exanimated and acquired under Olympus BX51 microscope. Oil Red O staining images were analyzed using Fiji ImageJ. The internal elastic lamina (IEL) boundaries were manually traced to define ROIs. Lipid-positive area was thresholded. The percentage of Oil Red O-positive area relative to total vessel area (IEL area) was calculated as lesion area % with background subtraction.

Real time quantitative polymerase chain reaction (PCR)

Total RNA was extracted from carotid arterial intima or HUVECs using Trizol (Invitrogen) according to

manufacturer's instruction. For carotid arterial intimal RNA, mice were anesthetized and euthanatized, followed by jugular vein cutting off. Pre-chilled PBS containing heparin was perfused from the left ventricle. After perfusion, both LCA and RCA were immediately isolated. 100ul Trizol were infused from one end of LCA and RCA using a 29G insulin syringe in a microfuge tube, while another ends were ligated with 6-0 silk sutures. After 60 seconds, the silk sutures were loosened, and the eluate were collected for intimal RNA extraction. cDNA was synthesized using FastQuant RT Kit (Tiangen). From cDNA template, real-time PCR amplification was performed using Hieff qPCR SYBR Green Master Mix and ABI Q6 real-time PCR equipment (Applied Biosystems) with primers listed in materials. Relative mRNA expression was determined by the threshold cycle values using comparative cycle threshold method and normalization with GAPDH.

Western blot analysis

HUVECs lysate were prepared in ice cold RIPA supplemented with PMSF and protease inhibitor cocktail. Cell debris were removed by centrifugation at 13000 rpm and 4°C for 20 minutes. Equal amount of lysates were subjected to SDS-polyacrylamide gel electrophoresis (PAGE) and transferred to PVDF membrane. After blocking with 5% (wt/vol) milk in TBS containing 0.1% (vol/vol) Tween-20, blots were incubated with appropriate primary antibodies diluted in TBST containing 1% (wt/vol) bovine serum albumin (BSA) overnight at 4°C and then corresponding HRP-conjugated antibodies diluted in TBST. The blots were developed by using Immobilon Western Chemiluminescent HRP Substrate (Millipore). Semi-quantification of proteins levels was analyzed using Image J software.

LC-MS/MS-based eicosanoids analysis

Eicosanoid mediators were extracted from 5×10^6 HUVECs subjected to steady flow or disturbed flow with 1 mL methanol/water solution (v/v=4:1) containing (d4) 13-HODE, (d4) 9-HODE, (d8) 15-HETE, (d8) 12-HETE and (d8) 5-HETE as the internal standards. After cell disruption and sonication for 5 minutes by a Bioruptor® Plus device (DIAGENODE S.A., BELGIUM), protein was precipitated in 4°C for 1 hour and then removed by centrifugation (12000 rpm, 4°C, 15 minutes). The upper-layer extract of each sample was vacuum-dried by a Labconco Vacuum Concentrator (Kansas, MO, USA) and then stored at -80°C before LC-MS/MS analysis.

The dry extracts were dissolved in 100 mL of methanol/water solution (v/v=1:4) and 10 mL of the

dissolved extract was injected for subsequent LC-MS/MS analysis. The eicosanoid analysis was carried out on a TSQ Quantiva Triple Quadrupole Mass Spectrometer equipped with an Ultimate 3000 UHPLC system (Thermo, MA, USA) according to the published method (Chen, et al. 2019)with modification. Eicosanoids were separated by an Acquity UPLC BEH C18 column (1.7 mm, 2.1×100 mm, Waters, MA, USA) maintaining at 40°C. The mobile phase A was 0.1% formic acid in water, and mobile phase B was 0.1% formic acid in acetonitrile. The flow rate was 0.3 mL/min, and the elution gradient was as follows: 0 - 0.5 min, 30% B; 1.0 - 2.5 min, 40% B; 4.5 - 6.5 min, 70% B; 9.0 - 12.0 min, 95%; 12.1 - 15.0 min, 30% B.

The MS with electrospray ionization was operated in negative ion mode with scheduled multiple reaction monitoring (MRM) for detection of eicosanoids and internal standards. Spray voltage was set at -2500 V. The sheath gas, aux gas, sweep gas, ion transfer tube and vaporizer temperature were set at 40 Arb, 8 Arb, 1Arb, 350°C and 350°C, respectively. The optimized MRM transitions and their respective collision energies were listed in Table 2.

Thermo Xcalibur Workstation and Quan Browser were employed for MRM data acquisition and processing. Quantification is based on the peak area ratio of measured eicosanoids and their corresponding internal standards with defined concentration.

Name	Formula	t_R (min)	Precursor Ion	Product Ion	Collision Energy	Internal Standard
13-HODE	$C_{18}H_{32}O_3$	6.66	295.3	195.2	18	(d4) 13-HODE
(d4) 13-HODE	$C_{18}H_{28}D_4O_3$	6.62	299.3	198.2	18	/
9-HODE	$C_{18}H_{32}O_3$	6.71	295.3	171.1	18	(d4) 9-HODE
(d4) 9-HODE	$C_{18}H_{28}D_4O_3$	6.68	299.3	172.1	18	/
15-HETE	$C_{20}H_{32}O_3$	6.8	319.3	219.2	14	(d8) 15-HETE
(d8) 15-HETE	$C_{20}H_{24}D_8O_3$	6.75	327.3	226.2	14	/
12-HETE	$C_{20}H_{32}O_3$	7.13	319.3	135.1	15	(d8) 12-HETE
(d8) 12-HETE	$C_{20}H_{24}D_8O_3$	7.08	327.3	184.2	15	/
5-HETE	$C_{20}H_{32}O_3$	7.44	319.3	115.1	17	(d8) 5-HETE
(d8) 5-HETE	$C_{20}H_{24}D_8O_3$	7.38	327.3	116.1	16	/
6k PGF1a	C20H34O6	2.43	369.3	163.1	25	(d4) 6k PGF1a

PGF2a	C20H34O5	3.45	353.3	309.3	19	(d4) 6k PGF1a
(d4) 6k PGF1a	C20H30D4O6	2.43	373.3	167.1	25	/
PGE2	C20H32O5	3.7	351.3	271.2	15	(d4) PGE2
(d4) PGE2	C20H28D4O5	3.69	355.3	275.2	17	/
11,12-EET	$C_{20}H_{21}O_3$	7.93	326.3	195.1	17	(d11)11,12-EET
(d11)11,12-EET	$C_{20}H_{21}D_{11}O_3$	7.87	337.3	197.1	16	/

Bioinformatics analysis

Transcription factor binding sites prediction was based on JASPAR database. The promoter region was defined as upstream of 2000bp to the transcriptional start site of ALOX15 gene.

Dual luciferase assay

HUVECs or 293T cell line were co-transfected with ALOX15 promoter-driven firefly luciferase reporter plasmid or and renilla luciferase plasmid by lipofectamine 2000 for 6 hours in Opti-MEM, followed by 42 hours incubation in fresh ECM medium. Luciferase activity was measured using the commercial Promega Dual-Luciferase™ Reporter (DLR™) Assay Systems kit according to the manufacturer's protocol. ALOX15 promoter activity was calculated as the ratio of bioluminescence intensity of firefly to renilla luciferase reporter.

Chromatin immunoprecipitation (ChIP)

Chromatin immunoprecipitation was performed using commercial kit with modification. Briefly, 37% formaldehyde was added to the medium of cultured cells to cross link DNA to protein for 10 minutes and quenched by glycine. Cells were collected by scrapping off and centrifuge at 800 × g for 5 minutes at 4 °C. Cross-linked proteins/DNA were released by cell lysis and sheared by sonication at 50% power 0.5 Hz for 8 minutes. ChIP-validated SREBP2 antibody or IgG was conjugated to ChIP protein A/G Magnetic Beads by 2 hours incubation at 4 °C on a rotating platform. Equal amount of sheared chromatins were then added to the antibody or IgG conjugated beads and incubated overnight at 4 °C on a rotating platform. The chromatin-antibody-beads complex and the input chromatin were digested with Proteinase K sequential incubation for 2 hours at 65°C and 15 minutes at 95°C. Supernatants containing the

immunoprecipitated DNA fragment were collected and used as the templet of real-time PCR for verification of ChIP enrichment. Primers used in real-time PCR are listed in materials.

Statistical analysis

Date is presented as mean ± SEM or median with interquartile range based on data distribution. Shapiro-Wilk test was used to test for data normality. Levene test was used to test for the equality of group variances. Proper post hoc tests were used to correct for multiple comparisons. Two-sided tests at 5% level of significance were used in all parametric and nonparametric tests. Detailed statistical methods were mentioned in corresponding figure legends. All statistical analyses were conducted using GraphPad Prism 9.0 or SPSS 26.0.

Reference

[1]. Chawengsub Y, Gauthier KM, Campbell WB. Role of arachidonic acid lipoxygenase metabolites in the regulation of vascular tone. *Am J Physiol Heart Circ Physiol* 2009;**297:** H495-507.

[2]. Chen GY, Zhang Q. Comprehensive analysis of oxylipins in human plasma using reversed-phase liquid chromatography-triple quadrupole mass spectrometry with heatmap-assisted selection of transitions. *Anal Bioanal Chem* 2019;**411:** 367-85.

[3]. Fernandes A, Hosseini V, Vogel V, Lovchik RD. Engineering solutions for biological studies of flow-exposed endothelial cells on orbital shakers. *PLoS One* 2022;**17:** e0262044.

[4]. Ghim M, Pang KT, Arshad M, Wang X, Weinberg PD. A novel method for segmenting growth of cells in sheared endothelial culture reveals the secretion of an anti-inflammatory mediator. *J Biol Eng* 2018;**12:** 15.

[5]. Li C*, et al.* CTRP5 promotes transcytosis and oxidative modification of low-density lipoprotein and the development of atherosclerosis. *Atherosclerosis* 2018;**278:** 197-209.

[6]. Li C, *et al.* Using En Face Immunofluorescence Staining to Observe Vascular Endothelial Cells Directly. *J Vis Exp* 2019.

[7]. Liu ZH, *et al.* C1q/TNF-related protein 1 promotes endothelial barrier dysfunction under disturbed flow. *Biochem Biophys Res Commun* 2017;**490:** 580-86.

[8]. McDuffie M*, et al.* Nonobese diabetic (NOD) mice congenic for a targeted deletion of 12/15-lipoxygenase are protected from autoimmune diabetes. *Diabetes* 2008;**57:** 199-208.

[9]. Nam D*, et al.* Partial carotid ligation is a model of acutely induced disturbed flow, leading to rapid endothelial dysfunction and atherosclerosis. *Am J Physiol Heart Circ Physiol* 2009;**297:** H1535-43.

[10]. Wang XQ*, et al.* Thioredoxin interacting protein promotes endothelial cell inflammation in response to disturbed flow by increasing leukocyte adhesion and repressing Kruppel-like factor 2. *Circ Res* 2012;**110:** 560-8.
